# Unveiling the Antibacterial Efficacy and Mechanistic Insights of MnO_2_ Nanoparticles for Advanced Therapeutic Applications

**DOI:** 10.3390/ijms26189104

**Published:** 2025-09-18

**Authors:** Istikhori Fitriannisa, Hanny Tika Draviana, Cheng-Pei Hsieh, Muhammad Saukani, Kai-Yi Tzou, Tsung-Rong Kuo

**Affiliations:** 1 International PhD Program in Biomedical Engineering, College of Biomedical Engineering, Taipei Medical University, New Taipei City 23564, Taiwan; d845112003@tmu.edu.tw (I.F.); d845112004@tmu.edu.tw (H.T.D.); 2 School of Biomedical Engineering, Taipei Medical University, New Taipei City 23564, Taiwan; b812112016@tmu.edu.tw; 3 Department of Mechanical Engineering, Faculty of Engineering, Universitas Islam Kalimantan MAB, Banjarmasin 70124, Kalimantan Selatan, Indonesia; saukani@uniska-bjm.ac.id; 4 Department of Urology, Shuang Ho Hospital, Taipei Medical University, New Taipei City 23561, Taiwan; 5 Department of Urology, School of Medicine, College of Medicine, Taipei Medical University, Taipei 11031, Taiwan; 6 Taipei Medical University Research Center of Urology and Kidney, Taipei Medical University, Taipei 11031, Taiwan; 7 Graduate Institute of Nanomedicine and Medical Engineering, College of Biomedical Engineering, Taipei Medical University, New Taipei City 23564, Taiwan

**Keywords:** MnO_2_ nanoparticle, antibacterial mechanism, reactive oxygen species, SEM analysis

## Abstract

Pathogenic bacterial infections pose serious health risks, underscoring the need for timely treatments. Manganese dioxide (MnO_2_) nanoparticles (NPs) have attracted considerable attention owing to their outstanding chemical stability, favorable biocompatibility, high reactivity, and catalytic ability to decompose hydrogen peroxide, making them promising antibacterial agents. A clear understanding of their antibacterial mechanisms is essential for evaluating their therapeutic potential in clinical settings. In this study, MnO_2_ NPs were synthesized by reacting potassium permanganate (KMnO_4_) with poly(allylamine hydrochloride) (PAH), ensuring complete conversion to MnO_2_ NPs. The resulting NPs were characterized for their physicochemical properties, and their antibacterial activity against *E. coli* and *S. aureus* was evaluated using growth curve assays and reactive oxygen species (ROS) quantification. Results indicated the killing efficiency of MnO_2_ NPs increased with exposure time and concentration, reflecting high susceptibility of both bacterial strains. Scanning electron microscopy (SEM) analysis revealed that the interaction between MnO_2_ NPs and bacterial cells caused significant disruption of cell wall integrity. This study provides a valuable platform for evaluating MnO_2_ nanoparticles as antibacterial agents and for exploring their mechanisms in medical applications.

## 1. Introduction

Pathogenic bacterial infections often cause widespread uncontrolled sickness and death and pose grave threats to human health [1,2,3]. These infections can attack various organs and tissues, necessitating effective and prompt treatment [4,5]. These common cases have been widely treated by antibiotics, a major 20th-century medical breakthrough with remarkable therapeutic success [6]. However, the improper or excessive use of antibiotics has led to adverse consequences, including the emergence of antibiotic-resistant complexes, where mutations in target proteins diminish their susceptibility and thereby reduce antibiotic efficacy [7,8,9,10,11]. Moreover, certain intrinsic characteristics of bacteria, such as their ability to produce toxins, can facilitate their colonization alongside other microbial species and contribute to the formation of resilient biofilms [11,12,13]. In the most severe cases, this can culminate in life-threatening conditions like sepsis, amputation, or even death [14,15]. According to 2019 statistics, bacterial infections caused by the six most prevalent pathogenic strains–*E. coli*, *S. aureus*, *K. pneumoniae*, *S. pneumoniae*, *A. baumannii*, and *P. aeruginosa*–were implicated in 929,000 deaths globally. The growing threat of antibiotic resistance has the potential to incite a worldwide pandemic or epidemic involving multidrug-resistant pathogens if timely and effective countermeasures are not implemented [16]. Paradoxically, the rate of discovery and development of novel antibiotics with innovative mechanisms of action has failed to keep pace with the alarming surge in bacterial infections and antibiotic resistance [17] due to the expensive and protracted nature of the process [18]. This disparity is evidenced by the fact that from 2010 to 2021, a mere 17 new antibiotics were successfully developed and licensed globally [19]. Consequently, there is an urgent imperative to formulate alternative therapeutic strategies and develop potent antibacterial agents to effectively combat these escalating threats.

Nanotechnology is defined as the scientific and engineering field that focuses on two key aspects: size-relatedness, as nanotechnology concentrates on the design and modification of shapes and sizes at the nanoscale; and novel nanotechnology which focuses on the discovery and development of solutions related to small entities [20] thereby harnessing the advantageous characteristics and properties of nanoscale materials. The synthesis, characterization, and application of designed nanomaterials or nanodevices are achieved through the integration of diverse academic disciplines, including biology, chemistry, physics, materials science, and medicine [21,22]. Certain applications of nanotechnology, such as drug/protein/gene delivery and immunological regulation, render it highly promising for developing therapeutic strategies, diagnostics, and vaccines [23,24]. Currently, nanotechnology is rapidly emerging as a promising large-scale approach for developing effective and safe antibacterial agents from a diverse array of compounds, offering versatile chemical and physical modification capabilities advantageous for treating bacterial infections and addressing antibiotic resistance [25]. The antibacterial properties of nanomaterials are bolstered by their substantial surface area, facilitating extensive contact with bacteria and thereby enhancing bioavailability, improving absorption, and providing a rapid pathway for drug delivery to target cells [26,27]. Among the various nanomaterials, nanoparticles (NPs) have attracted global interest for their unique physical properties, enabling broad applications in biology, electronics, sensing, and optoelectronics [28]. Unlike conventional antibacterials or antifungals, their nanoscale size allows efficient penetration of microorganism cell walls and membranes, a crucial factor in their antimicrobial efficacy.

Among the myriad metal oxide NPs, manganese dioxide (MnO_2_) NPs stand out as one of the most extensively utilized nanomaterials in industrial applications, owing to their remarkable properties, including high reactivity, diverse morphologies, natural abundance, and cost-effective production [29,30,31,32,33,34]. With their unique properties, including controllable particle size, great chemical stability, amazing biocompatibility, and environmental friendliness [35,36,37,38,39]. MnO_2_ NPs have been widely exploited and rigorously investigated in many biomedical sectors, including antibacterial, antifungal, antibiofilm, antioxidant, and anticancer applications [40]. Research conducted by Ikram et al. on triplex-based nanomaterials, synthesized through a co-precipitation process, demonstrated the efficacy of Mo/chitosan (CS)-doped MnO_2_ NPs in eliminating pollutants from wastewater through their robust scavenging capabilities and antibacterial properties [30]. On the other hand, Lu et al. investigated biogenic synthesized *Viola betonicifolia* extract (VBLE)-MnO_2_ NPs, which determined notable antibacterial, antifungal, and biofilm-inhibitory activities against a range of microbial species and displayed notable antioxidant potential [28]. Another study from Prasad et al. reported the synthesis of bioinorganic A-MnO_2_ NPs composed of a polyelectrolyte–albumin complex and MnO_2_, demonstrating their capacity to downregulate tumor-associated factors, thereby inhibiting tumor growth and promoting cancer cell death [33]. The antifungal properties of MnO_2_ were investigated by Faisal et al. against four strains of fungi responsible for apple spoilage, while Alarfaj et al. also examined MnO_2_ derived from *Malus domestica* peel extract to detect cephalexin (CPX) antibiotics [41,42]. It is well-established that manganese is well-recognized as an essential element and activator of numerous vital enzymes in the human body [21] and manganese-based compounds have garnered significant attention due to their low potential cytotoxicity, positioning them among the most frequently used metal elements in the development of disease treatment approaches [43,44]. MnO_2_ NPs can catalyze the decomposition of hydrogen peroxide, generating oxygen and releasing manganese ions (Mn^2+^) under both acidic and physiological conditions (pH 7.4) [45,46]. Consequently, MnO_2_ NPs can continuously generate oxygen and alleviate oxidative stress, thereby mitigating inflammation associated with infections [47]. Capitalizing on their critical functions in human health, manganese-based NPs have emerged as promising candidates for novel diagnostic strategies, vaccine development, and drug formulations [21].

In this study, we investigated the therapeutic potential and killing mechanisms of MnO_2_ NPs. The physicochemical properties of the MnO_2_ NPs were confirmed through characterization techniques including ultraviolet-visible (UV-Vis) spectroscopy, X-ray diffraction (XRD) spectroscopy, Raman spectroscopy, transmission electron microscopy (TEM), high-resolution TEM (HR-TEM), scanning electron microscopy (SEM) and energy-dispersive X-ray (EDX) spectroscopy. Antibacterial activity was assessed via bacterial growth curve analysis, while intracellular reactive oxygen species (ROS) generation was quantified to evaluate oxidative stress induced during treatment. Additionally, the antibacterial mechanisms of MnO_2_ NPs were elucidated through direct visualization using scanning electron microscopy (SEM).

## 2. Results

### 2.1. Physicochemical Characterization of MnO_2_ NPs

**Figure 1:**
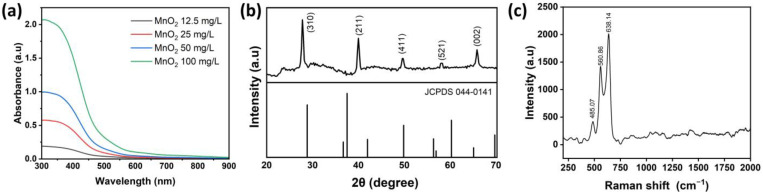
Overview of MnO_2_ NPs characterization: (**a**) UV-Vis absorption results, (**b**) XRD analysis, and (**c**) Raman spectra.

MnO_2_ NPs were prepared using a one-pot system method. MnO_2_ NPs characteristics were assessed through analyzing absorption peaks using UV-Vis spectroscopy, as illustrated in Figure 1a. Various concentrations of MnO_2_ NPs were also analyzed to investigate the concentration’s impact on absorption. Results depict that with an increasing concentration, absorbance values also proportionally increased. Based on Figure 1a, it is evident that the highest peaks of MnO_2_ NPs were around 391 nm at a concentration of 12.5 mg/mL, 373.5 nm at 25 mg/mL, 363 nm at 50 mg/mL, and 355.5 nm at 100 mg/mL. Moreover, an XRD analysis was conducted to identify crystalline structures of MnO_2_ NPs, its composites, and impurities. Figure 1b illustrates the XRD pattern of MnO_2_ NPs. The XRD analysis of MnO_2_ NPs closely matched the standard reference α-MnO_2_ (JCPDS No. 044-0141). Peaks that closely aligned with the standard reference exhibited 2θ values at 27.791, 39.9995, 49.563, 58.109, and 65.841, respectively, corresponding to the (310), (211), (411), (521), and (002) crystallographic planes. The local structure and phase composition of MnO_2_ NPs were further analyzed using Raman spectroscopy, as depicted in Figure 1c. As shown in Figure 1c, there were three bands of MnO_2_ NPs at 485.07, 560.86, and 638.14 cm^−1^. Three Raman bands located at 485.07, 560.86, and 638.14 cm^−1^ in MnO_2_ NPs exhibited values akin to the vibrational characteristics of birnessite-type MnO_2_ NPs as previously reported [48,49].

**Figure 2:**
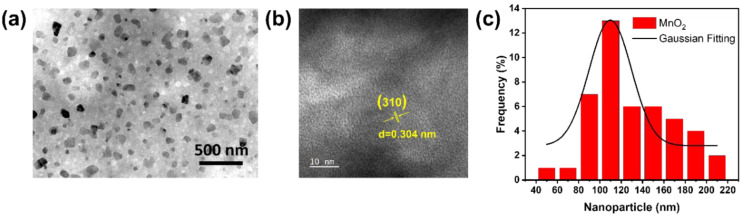
(**a**) TEM observation, (**b**) HR-TEM observation, and (**c**) size distribution of MnO_2_ NPs.

TEM imaging was used for further characterization to examine the topography and average particle size distribution of MnO_2_ NPs. Based on Figure 2a, MnO_2_ NPs had an approximate size of 100 nm. In addition, HR-TEM was conducted to validate the dimensions and morphology of MnO_2_ NPs. As depicted in Figure 2b, lattice fringe patterns of MnO_2_ NPs exhibited a d-spacing of 0.304 nm, aligning with the (310) plane of MnO_2_ NPs as referenced (JCPDS No. 044-0141). The average size was further validated using a Gaussian fitting curve simulation. This was evidenced by the histogram graph depicting the size distribution of MnO_2_ NPs based on 45 NPs present in the TEM image in Figure 2a. As shown by the Gaussian fitting curve in Figure 2c, the average particle size of MnO_2_ NPs was approximately 109.7 ± 5.6 nm. Previous studies have confirmed that MnO_2_ nanoparticles exhibit excellent stability in deionized (DI) water, showing no aggregation [50] and no significant changes in particle size or zeta potential [51].

**Figure 3:**
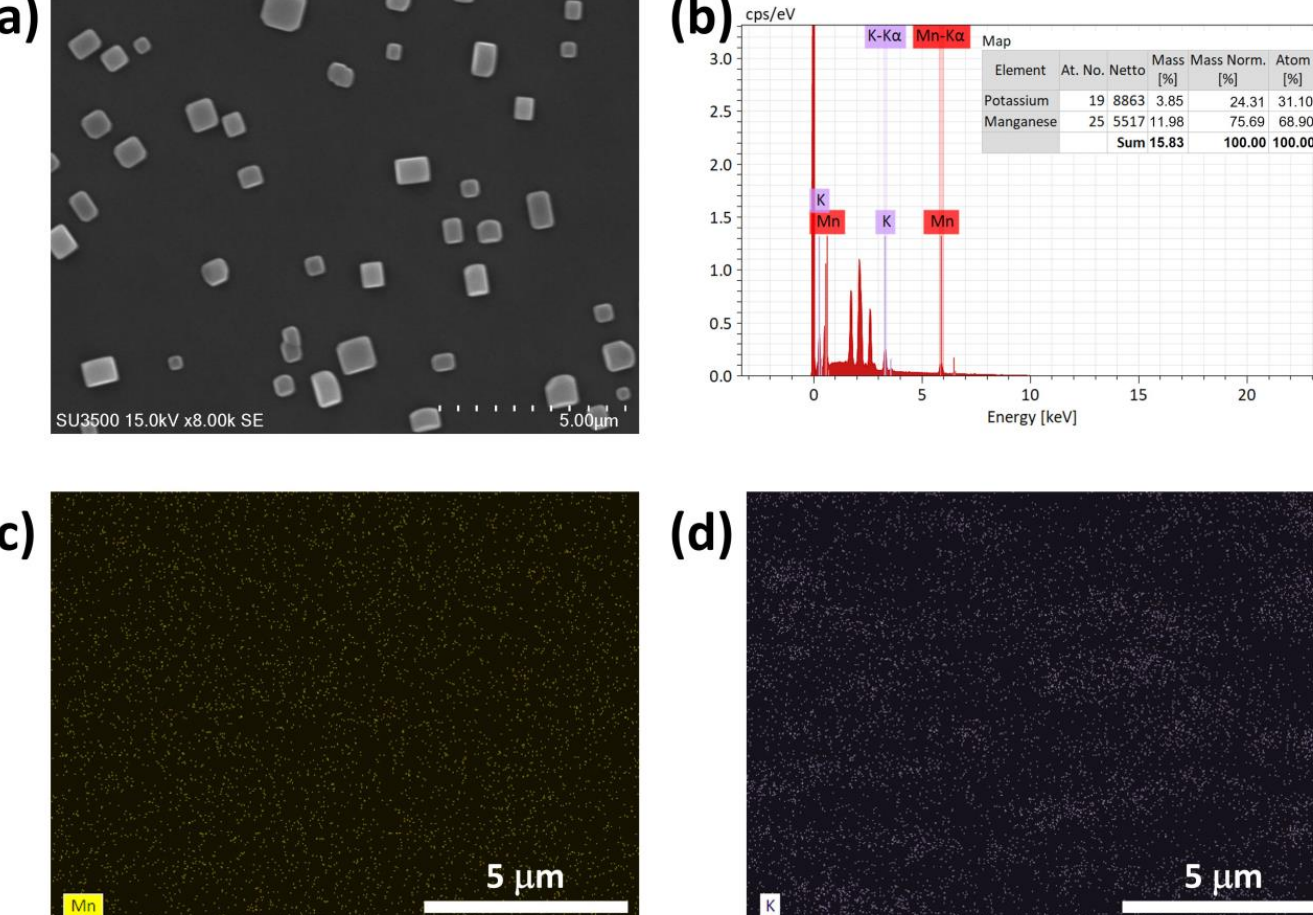
(**a**) SEM images, (**b**) EDX analysis, and (**c**,**d**) EDX mapping of MnO_2_ NPs.

To understand surface properties and morphology of MnO_2_ NPs using SEM as depicted in Figure 3a, the crystal structure of MnO_2_ NPs appeared as cube-like particles with a smooth surface. Further analysis of SEM images was conducted to identify the EDX elemental distribution of MnO_2_ NPs. As illustrated in Figure 3b, MnO_2_ NPs consisted of potassium (K) at 24.31% and manganese (Mn) at 75.69%. The elemental distribution map of MnO_2_ NPs is also depicted in Figure 3c.

### 2.2. Antibacterial Study of the Synthesized MnO_2_ NPs

**Figure 4:**
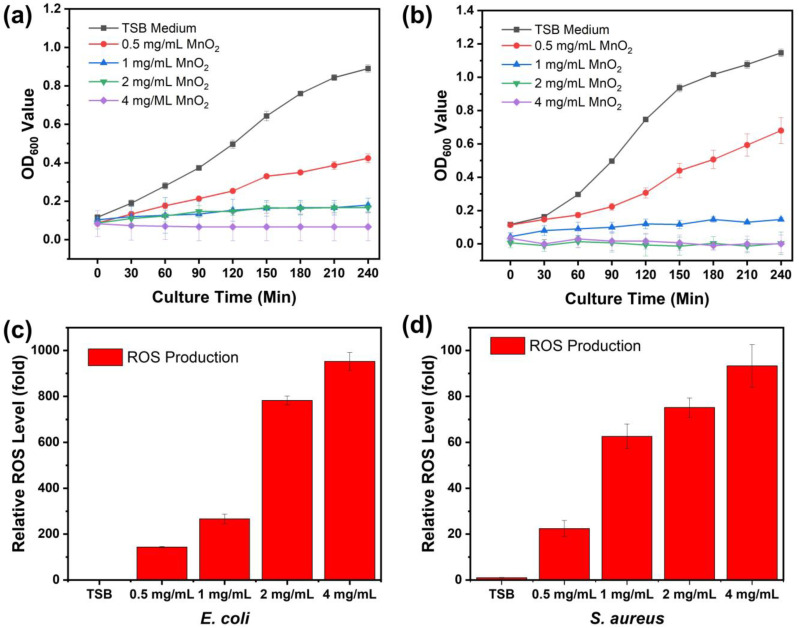
Antibacterial activity and ROS generation of *E. coli* (**a**,**c**) and *S. aureus* (**b**,**d**).

A bacterial growth curve analysis was conducted to assess impacts of various concentrations MnO_2_ NPs on the growth of Gram-negative bacteria (*E. coli*) and Gram-positive bacteria (*S. aureus*). Both *E. coli* and *S. aureus* were activated and then were incubated at 37 °C. OD values of the bacterial solutions were monitored every 30 min for 240 min. The minimum inhibitory concentration of MnO_2_ NPs was obtained through the growth curves assay. From Figure 4a,b, the MIC values of MnO_2_ NPs against *E. coli* and *S. aureus* was determined to be 1 mg/mL, at which no bacterial growth occurred. As shown by the bacterial growth presented in Figure 4a,b, OD_600_ values of both *E. coli* and *S. aureus* decreased as the concentration of MnO_2_ NPs increased. OD_600_ values for the controls of these two groups represented by *E. coli* and *S. aureus* in TSB solution medium with no treatment were ~0.89 and ~1.147, respectively, after incubation for 240 min. Furthermore, OD_600_ values for *E. coli* treated with concentrations of 0.5, 1, 2, and 4 mg/mL of MnO_2_ NPs were 0.4, 0.18, 0.17, and 0.067, respectively. OD_600_ values for *S. aureus* treated with same concentrations were 0.68, 0.147, 0.003, and 0, respectively. Respective ROS levels of *E. coli* treated with serial concentration 0.5, 1, 2, and 4 mg/mL of MnO_2_ NPs were 143.31-, 266.21-, 782.39-, and 952.64-fold (Figure 4c). ROS levels of *S. aureus* at the same concentrations of treated MnO_2_ NPs were 22.42-, 62.66-, 75.13-, and 93.29-fold (Figure 4d), respectively.

### 2.3. Interaction Observations and Bacterial Death Mechanism Induced by Synthesized MnO_2_ NPs

**Figure 5:**
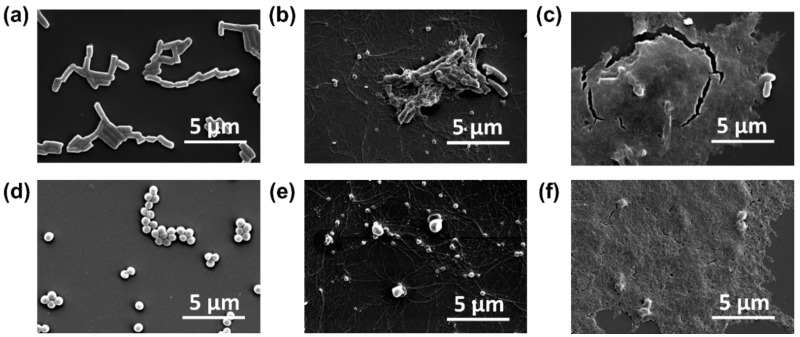
Scanning electron microscopy (SEM) micrograph of *E. coli* (**top columns**) and *S. aureus* (**bottom columns**), without treatment (**a**,**d**) and treated with 2 mg/mL (**b**,**e**) and 4 mg/mL MnO_2_ NPs (**c**,**f**).

SEM observation results revealed the interaction of MnO_2_ NPs with the two bacterial strains, *E. coli* and *S. aureus*. This analysis unveiled compelling evidence of the antibacterial activity of MnO_2_ NPs against these bacteria and elucidated how NPs treatment induced bacterial damage, leading to cell death. Figure 5a,d depict untreated bacterial conditions, which served as controls for this analysis. Both bacterial species exhibited normal morphologies, with *E. coli* displaying a solid rod-like shape and *S. aureus* exhibiting a spherical shape. Stark differences between the bacterial morphologies before and after treatment were evident. Upon treatment with 2 mg/mL MnO_2_ NPs as depicted in Figure 5b,e, the bacteria underwent morphological alterations, including cell membrane disruption, cellular defects and irregularities, bacterial leakage, and the formation of MnO_2_ NPs agglomerates. This agglomeration intensified with an increased MnO_2_ NPs concentration of 4 mg/mL, as illustrated in Figure 5c,f. The dense background observed in these images corresponds to MnO_2_ NPs agglomerates, which tended to accumulate around the bacterial cells. Treatment with this concentration resulted in bacterial entrapment within the MnO_2_ NPs agglomerates, leading to significant structural damage and fragmentation, ultimately causing bacterial destruction and death.

**Figure 6:**
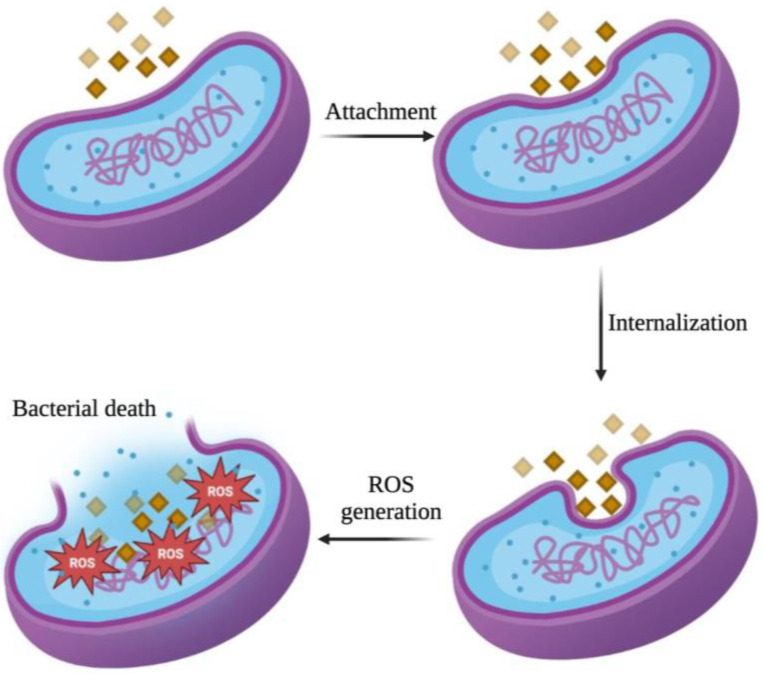
Illustration of antibacterial mechanism of MnO_2_ NPs against bacterial cells.

Bacteria possess cell membranes and plasma membranes that function as barriers, separating the bacteria from the external environment and preventing nearly all potential damage that may occur to the cell. Although Gram-positive and Gram-negative bacteria exhibit differences in their cell membrane structure, thickness, and composition, their primary role in self-defense is consistent. The interaction between metal oxide NPs and bacteria induces alterations in the bacterial cell surface, which initiates the antibacterial activity of these NPs. This study demonstrated that the NPs concentration affected their antibacterial activity, consistent with findings of a study by Ogunyemi et al. [38] on the concentration-dependent antibacterial activity of NPs. Furthermore, the nanoscale structure of NPs (1–100 nm) confers a distinct advantage to their antibacterial mechanism, facilitating their attachment and internalization process into the micron-sized bacteria, and causing cellular morphological damage and distortions which cause the death of bacterial cells (Figure 6) [52,53,54,55].

## 3. Discussion

The synthesis of MnO_2_ nanoparticles is typically carried out using polymers that serve a dual function: reducing potassium permanganate (KMnO_4_) to MnO_2_ and enhancing the colloidal stability of the product [56]. In this study, the cationic polymer poly(allylamine hydrochloride) (PAH) was employed not only as a reducing agent but also as a polyelectrolyte that acts as a protective stabilizer for the resulting colloidal NPs [33,46,57]. KMnO_4_ is a strong oxidant and its direct redox reaction with PAH proceeds spontaneously, yielding a dark brown MnO_2_ NPs colloidal suspension [58,59]. The intrinsic positive charge of PAH interacts electrostatically with the negatively charged surface of MnO_2_, resulting in the well-stable MnO_2_ NPs and imparting a positive surface charge to the final dispersion [33,57,58,60]. The synthesis was performed in aqueous solution at room temperature through a one-pot synthesis approach. This method offers several advantages, including rapid execution, high reproducibility, and the generation of a stable MnO_2_ NPs due to the protective stabilizer layer through electrostatic repulsion by the PAH [61]. In this study, the one-pot synthesis provided a straightforward and cost-effective route [62,63] without the need for other additional reducing agents or protective surfactants to ensured colloidal stability [57,64]. Beyond its simplicity, this method represents an effective strategy to enhance reaction efficiency within a single vessel, thereby minimizing chemical waste and saving time, particularly when the same reagents are utilized for subsequent reactions [65]. Furthermore, this protocol suppressed the formation of byproducts, resulting in a high yield of the desired nanoparticles product.

For physicochemical characterization, the UV-Vis absorption peaks were associated with the d-d transition of Mn ions within MnO_2_ NPs. This phenomenon may be ascribed to electrons being excited from the valence band into the conduction band induced by photon absorption [66,67]. In line with these electron transitions, the UV-Vis spectra of MnO2 NPs exhibited absorption intensity in a concentration-dependent manner, while the peaks position remained at consistent wavelengths without observable wide shifts, indicating the overall intensity scaled proportionally with the nanoparticle concentration. This result suggests that the NPs have good colloidal stability and minimal aggregation in dispersion. Based on the XRD analysis, the presence of distinct diffraction peaks in the visual representation indicated the substantial crystallinity of MnO_2_ NPs [68,69]. The Raman band observed at the peak of 638.14 cm^−1^ was attributed to the stretching vibration v2 (Mn-O) of the MnO_6_ group. The band with a peak at 560.86 cm^−1^ was correlated with the v3 (Mn-O) stretching vibration in the basal plane of [MnO_6_] sheets. Meanwhile, the band with a peak at 485.07 cm^−1^ was interpreted as the Mn-O-Mn bending vibrations within the MnO_2_ octahedral lattice. The successful preparation of MnO_2_ NPs was confirmed through detailed physicochemical characterization, highlighting their distinct electronic transitions, substantial crystallinity, and characteristic vibrational modes.

The analysis of OD_600_ values for *E. coli* and *S. aureus* demonstrated that the killing efficiency of MnO_2_ NPs increased with both time and concentration. Growth curve analysis indicated that the inhibition of both *E. coli* and *S. aureus* began at a concentration of 1 mg/mL, which can be defined as the minimum inhibitory concentration (MIC). While at 4 mg/mL, complete bacterial killing occurred as the OD_600_ values of both *E. coli* and *S. aureus* dropped to nearly zero. Nevertheless, it was concluded that both *E. coli* and *S. aureus* exhibited high vulnerability to treatment with MnO_2_ NPs. The antibacterial activity itself related to the nano-structured materials, which if compared to their bulk counterparts, exhibits greater potency due to the high surface-to-volume ratio, and it affects a significant increase in the surface area as reaction sites [70,71,72]. In this study, the antibacterial mechanism of MnO_2_ NPs can be defined into complementary processes: (1) the release of Mn^2+^ ions that interact with sulfhydryl/thiol groups in proteins, leading to enzyme inactivation and disruption of membrane function; (2) the generation of ROS, which disrupts metabolic processes, inhibits respiratory enzymes, and induces oxidative stress; and (3) membrane disruption and leakage of cell contents. In the initial phase, MnO_2_ NPs interact with the bacterial membrane through non-covalent forces, including electrostatic interactions, hydrophobic interactions, van der Waals forces, and receptor-ligand interactions. These physicochemical interactions facilitate strong attachment to negatively charged bacterial surface components—teichoic acids in Gram-positive species and lipopolysaccharides in Gram-negative species—thus prolonging the retention of nanoparticles on the membrane [73]. Smaller MnO_2_ nanoparticles, owing to their larger specific surface area, can more readily penetrate bacterial cells and induce damage to intracellular components by disrupting the membrane cell and subsequent loss of cellular contents [74]. When Mn^2+^ ions contact with bacterial cell membranes, they can reduce the membrane dipole potential and alter the hydration state of the phospholipid headgroups. These changes alter the net surface charge of the membrane, causing local disruption of the membrane structure and increasing permeability [36,73,75].

In parallel, the dissolution of MnO_2_ can release Mn^2+^ ions that interact with bacterial proteins, particularly at sulfhydryl (–SH) groups, forming stable S—Mn bonds that compromise enzymatic function and metabolic activity. The mechanism involves the replacement of H^+^ ions from sulfhydryl or thiol groups by Mn ions within biological macromolecules, which ultimately results in protein inactivation, disruption of membrane permeability, and subsequent cell death [76]. In addition to possible intracellular interactions, the generation of ROS also substantially contributed to the observed antibacterial performance of NPs in this study [77,78]. This phenomenon is due to the ability of NPs to disrupt cellular metabolic processes, block respiratory enzymes, halt bacterial proliferation, and promote the rise in ROS. Furthermore, the generation of ROS heightens oxidative stress in cells, ultimately resulting in DNA, protein, and cellular structural damage, which lead to leakage of organelles and cytoplasmic materials and cell death [38]. This was also evident by the ROS analysis which examined intracellular ROS production absorption in *E. coli* and *S. aureus* using DCFH-DA as an ROS indicator. ROS levels from both bacteria indicated high absorbance intensities, especially those produced from treated *E. coli*. The restrained and lack of bacterial development observed in growth curves of both bacterial types resulted from the high amount of ROS levels present in both bacterial populations. SEM observations provide robust support that the interaction between MnO_2_ NPs and both bacterial strains exerted antibacterial activity by compromising the integrity of bacterial cell walls, highlighting the potent antibacterial properties of MnO_2_ NPs against the targeted bacterial species, encompassing both Gram-positive and Gram-negative bacteria. Consistent with these processes, our SEM analysis revealed significant changes in bacterial cell morphology, including membrane rupture and leakage of bacterial cellular contents, particularly at higher concentrations of MnO_2_ NPs, which exhibited extensive agglomeration. The combination of surface interaction, physical entrapment, oxidative stress from ROS production, and protein inactivation from Mn^2+^ ions work together to destroy bacterial cell integrity and kill the cells completely.

In accordance with previous studies, MnO_2_ NPs demonstrate antibacterial properties through a mechanism that involves membrane disruption and the production of reactive oxygen species (ROS). A study by Liu et al. (2021) demonstrated that Mn-based OA-MnO_2_ nanozymes effectively generated ROS capable of eradicating Gram-positive *S. aureus* and Gram-negative *E. coli* bacteria [63]. According to Corrales et al. (2022), after 20 min of treatment with MnO_2_ under dark conditions, ~1.8 log units of the bacterial population survived [79]. This indicates the presence of bactericidal activity, which is likely due to the destruction of bacterial lipid molecules and cell walls, as well as the resulting oxidative stress from the generation of ROS. Ikram et al. (2022) also reported that Mo/Cs-doped MnO_2_ exhibited broad-spectrum antibacterial efficacy against *S. aureus* and *E. coli*, with inhibition zones expanded as Mo concentration increased [30]. This increase in activity was associated with the formation of ROS and radical entities that interact with genomic structures and the outer walls of bacterial microbes. Although studies reporting MIC values against *E. coli* and *S. aureus* are limited, in our study, inhibition of *E. coli* and *S. aureus* growth began at 1 mg/mL MnO_2_ NPs (MIC), with complete inhibition at 4 mg/mL. In addition, SEM observation consistently demonstrated morphological evidence of cell membrane disruption following MnO_2_ NP treatment. Even though direct comparisons of MIC values remain limited, research has consistently shown how important oxidative stress and surface interaction are in mediating antibacterial activity.

Our findings further support the potential of MnO_2_ NPs as antibacterial agents for fighting bacterial infections for further research. The results, which were demonstrated through growth inhibition analysis followed by analysis of ROS generation—a key component of the antibacterial reaction mechanism—and observation of bacterial morphology through SEM analysis, contribute to the limited evidence supporting MnO_2_ NP research. Compared to traditional antibiotics, which act on a single molecular target and lead to rapid bacterial resistance, MnO_2_ exhibits antibacterial properties through multiple mechanisms, highlighting its potential as an alternative to traditional antibiotics in combating bacterial infection [80,81]. Despite this, additional limitations for future therapeutic applications remain inadequately investigated, including colloidal stability, biosafety, biodegradability, and biocompatibility. The short- and long-term effects on humans significantly hinder clinical approval. Although excessive Mn accumulation in humans may enhance neurotoxicity, MnO_2_ NPs exhibit significant therapeutic potential. Consequently, subsequent research must focus on surface modification, controlling nanoparticle stability, cytotoxicity assessment in mammalian cells, the evaluation of biocompatibility, in vivo antibacterial efficacy, and biosafety in humans to be useful for various biomedical applications [82].

## 4. Materials and Methods

### 4.1. Materials

Potassium permanganate (KMnO_4_) was obtained from Acros Organics™ (Thermo Scientific™, Taipei, Taiwan). Poly(allylamine hydrochloride) (PAH) and 2′,7′-dichlorofluorescein diacetate (DCFH-DA) were supplied from Sigma-Aldrich (St. Louis, MO, USA). Ethanol dehydrate was supplied by Bioman Scientific (Taipei, Taiwan), while Hoechst 33342 was obtained from Bio-Genesis Technologies (Taipei, Taiwan). Kanamycin and Luria–Bertani (LB) broth (Miller) were purchased from BioShop (Burlington, ON, Canada), and tryptic soy broth (TSB) was acquired from Condalab (Madrid, Spain). Deionized water (DI water) was provided by the Core Facility Center of the College of Biomedical Engineering, Taipei Medical University (Taipei, Taiwan).

### 4.2. Preparation of MnO_2_ NPs

MnO_2_ NPs samples were prepared by directly mixing 66.4 mg of potassium permanganate (KMnO_4_) in 18 mL of deionized water (DI water) with 77 mg of poly(allylamine hydrochloride) (PAH) dissolved in 2 mL of DI Water. Each solution was stirred independently until complete dissolution was achieved. Afterward, both solutions were mixed and stirred steadily for 15 min at room temperature. 1 mL of the mixture was placed in a bottle and dried in an oven overnight. After drying, the bottle was weighed to calculate the MnO_2_ NPs concentration. The MnO_2_ NPs samples were then characterized by UV-Vis spectrometer (Jasco-V770, Sunway Scientific Corporation, Tokyo, Japan), X-ray diffraction (XRD) spectrometer (Bruker, Billerica, MA, USA with Cu Kα radiation, generated at 30 mA and 30 kV), and Raman spectrometer (UniDRON Laser Spectroscopy Confocal Micro Raman Spectrometer, CLT, New Taipei City, Taiwan). Approximately 20 μL (1 drop) of MnO_2_ NPs samples is dripped onto the copper grid and oven-dried for 2–3 min, then imaged by transmission electron spectroscopy (TEM) (HT-7700, Hitachi, Tokyo, Japan) and high-resolution (HR)-TEM (JEM-2100F, JEOL Ltd., Tokyo, Japan). Similarly, 20 µL of the sample was pipetted onto a silica plate and subsequently imaged by scanning electron microscopy (SEM) (SU-3500, Hitachi, Tokyo, Japan) and energy-disperse X-ray (EDX) spectrometer (Quantax EDS, Bruker, Billerica, MA, USA).

### 4.3. Evaluation of the Antibacterial Activity of MnO_2_ NPs

The antibacterial activity of MnO_2_ nanoparticles was evaluated against *Escherichia coli* and *Staphylococcus aureus*. Growth medium was prepared by dissolving 6 g of TSB powder in 200 mL of sterile water and sterilizing the solution in an autoclave at 121 °C for 1 h. Following sterilization, the medium was cooled to room temperature. Frozen bacterial stocks were revived by inoculating 20 µL of each strain into 3 mL of TSB, which was incubated overnight at 37 °C in a shaker (LM-80DR, YIH DER Technology, New Taipei City, Taiwan) at 170 rpm. Successful revival and growth were confirmed the next day by measuring optical density at 600 nm (OD_600_) using a cell density meter (Biochrom Ultraspec 10, Holliston, MA, USA). To assess antibacterial efficacy, MnO_2_ nanoparticles were added at several concentrations of 0.5, 1, 2, and 4 mg/mL to 1.5 mL aliquots of bacterial culture. The cultures were incubated for 4 h at 37 °C under shaking (170 rpm), and OD_600_ readings were recorded every 30 min. Growth curves were subsequently analyzed to determine the impact of each nanoparticle concentration on bacterial viability.

### 4.4. Intracellular ROS Production Measurements

To assess the intracellular concentration of ROS generated by bacteria, DCFH-DA dye was employed. DCFH-DA can be transformed into 2′,7′-dichlorofluorescein (DCF), and its intensity was quantified using a fluorospectrometer with λ_exc_/λ_em_ set to 488/525 nm. Hoechst 33342, which has excitation/emission peaks at 350/461 nm, was employed to estimate the total bacterial population. For ROS detection, 1.5 mL of bacterial culture with an OD_600_ of 0.1 was treated with MnO_2_ NPs at concentrations of 0.5, 1, 2, and 4 mg/mL. The mixtures were incubated at 37 °C and 170 rpm for 4 h, followed by the addition of 0.4 µL each of DCFH-DA and Hoechst 33342. The mixtures were incubated in the dark for 30 min and subsequently centrifuged at 10,000 rpm for 2 min. The precipitations were resuspended in 400 µL of sterile water, and 300 µL aliquots were transferred to a black 96-well plate. Fluorescence was recorded using a microplate reader (Varioskan Flash Multimode Reader, Thermo Scientific). ROS levels were normalized to bacterial count, and the relative ROS production was compared across all tested nanoparticle concentrations and the control group.

### 4.5. Preparation of Bacterial Sample Treated with MnO_2_ NPs for SEM Observation

For SEM observation, 1.5 mL of a bacterial suspension composed of *E. coli* and *S. aureus* (OD_600_ = 0.2) was mixed with 1.5 mL of MnO_2_ NPs solutions at 0.5 and 4 mg/mL. The mixtures were incubated for 4 h. After incubation, the samples were placed into 1.5 mL microcentrifuge tubes and spun at 3500 rpm for 3 min. The supernatant was carefully removed, and the samples underwent were washed three times with DI water. 4% paraformaldehyde for 500 µL was gently added to fix the cells, continued by another three washes with DI water. The samples were then centrifuged again at 2500 rpm for 3 min and treated with 70% ethanol. Finally, a 20 µL drop of the treated sample was placed onto a silica substrate and prepared for SEM imaging.

## 5. Conclusions

MnO_2_ NPs were prepared via a simple one-pot method by directly mixing potassium permanganate (KMnO_4_) and poly(allylamine hydrochloride) in deionized water. The physicochemical properties of MnO_2_ NPs were confirmed through characterization using UV-Vis spectroscopy, Raman spectroscopy, TEM, HR-TEM, and EDX spectroscopy. The antibacterial activity was assessed through bacterial growth curves, which showed that the effectiveness of MnO_2_ nanoparticles against Gram-negative (*E. coli*) and Gram-positive (*S. aureus*) bacteria increased with higher NPs concentrations. The generation of intracellular ROS caused by these NPs inside the cells also substantially contributed to their observed antibacterial performance. In summary, the mechanisms of action of MnO_2_ NPs involved their adherence and penetration into cells facilitated by their nanoscale dimensions, which caused distortions of cell structures and interfered with cellular metabolic activities, triggering the rise in intracellular ROS levels. This consequently led to cellular structural damage, causing bacterial cell leakage and ultimately cell death.

## Data Availability

The data presented in this study are available upon request from the corresponding author.

## Abbreviations

The following abbreviations are used in this manuscript:

MnO_2_	Manganese dioxide
NPs	Nanoparticles
KMnO_4_	Potassium permanganate
PAH	Poly(allylamine hydrochloride)
ROS	Reactive oxygen species
UV-Vis	Ultraviolet-visible
TEM	Transmission electron microscopy
SEM	Scanning electron microscopy
DCFH-DA	2′,7′-Dichlorofluorescein
LB	Luria–Bertani
TSB	Tryptic soy broth
DI	Deionized
